# Perturbing *O*-GlcNAcase Modulates the Expression and Distribution of Galectin-3

**DOI:** 10.3390/cells15131181

**Published:** 2026-06-29

**Authors:** Mana Mohan Mukherjee, Asmita Pramanik, Marcella Kolodrubetz, Devin Biesbrock, Kenneth A. Jacobson, John A. Hanover

**Affiliations:** 1 Cell Biochemistry Section, Laboratory of Cell and Molecular Biology, National Institute of Diabetes Digestive and Kidney Disease (NIDDK), National Institutes of Health, Bethesda, MD 20892, USA; marcyc@intra.niddk.nih.gov (M.K.); devinbiesbrock@gmail.com (D.B.); 2 Molecular Recognition Section, Laboratory of Bioorganic Chemistry, National Institute of Diabetes Digestive and Kidney Disease (NIDDK), National Institutes of Health, Bethesda, MD 20892, USA; asmita.pramanik@nih.gov (A.P.); kennethj@niddk.nih.gov (K.A.J.)

**Keywords:** *O*-GlcNAcase, galectin-3, *Oga* KO mice, tissue, blood, obesity, type 2 diabetes

## Abstract

**Highlights:**

Galectin-3 (Gal-3) is a multifunctional lectin with emerging roles in obesity, diabetes, and chronic inflammatory diseases. However, the pathways controlling its expression remain largely undefined. Here, we demonstrate that high-fat-diet-induced obesity elevates circulating Gal-3 in mice, while loss of *O*-GlcNAcase (*Oga*) activity leads to a systemic reduction in Gal-3 expression. These findings uncover a previously unrecognized regulatory axis connecting the *O*-GlcNAc cycling enzyme, *Oga*, to Gal-3 production, suggesting that *Oga*-dependent signaling influences the inflammatory and metabolic roles of Gal-3. Understanding this relationship can provide new insight into how nutrient sensing and post-translational modification pathways intersect to modulate biomarkers of metabolic disease.

**What are the main findings?**

- Circulating Gal-3 concentration is upregulated in HFD-induced obese and type 2 diabetic mice.
- Circulating Gal-3 concentration is significantly downregulated in *Oga* KO mice.

**What is the implication of the main finding?**

- Tissue distribution and blood Gal-3 concentration is a function of *Oga* expression.
- Circulating Gal-3 levels are not solely determined by glucose availability but are critically influenced by *Oga* expression.

**Abstract:**

Galectin-3 (Gal-3) is a β-galactoside-binding lectin implicated in metabolic inflammation, cardiovascular and renal dysfunction, neurodegenerative disorders, and obesity-related pathologies. Although Gal-3 is recognized as a clinically relevant biomarker, the mechanisms controlling its tissue expression and circulating abundance remain poorly defined. *O*-GlcNAcase (*Oga*; encoded by Mgea5), the enzyme that removes *O*-linked β-*N*-acetylglucosamine (*O*-GlcNAc) from proteins, regulates nutrient-sensitive signaling and transcriptional processes that overlap with Gal-3 associated disease pathways. To investigate the relationship between metabolic status and Gal-3 expression, male mice were fed a high-fat diet (HFD) for eight weeks to induce obesity. HFD-fed mice exhibited significant increases in body weight and fasting and fed blood glucose levels compared with lean controls, confirming metabolic dysregulation. ELISA revealed approximately threefold higher serum and plasma Gal-3 concentrations in obese mice, indicating enhanced Gal-3 production in diet-induced obesity. To determine whether *Oga* regulates Gal-3 expression, *Oga* wild-type (WT), heterozygous (HET), and knockout (KO) mice were analyzed. Circulating Gal-3 protein levels were significantly reduced in *Oga* KO mice, with intermediate levels in *Oga* HET animals. RT-qPCR revealed genotype-dependent modulation of Gal-3 (*Lgals3*) mRNA expression across multiple tissues, demonstrating tissue-specific regulation by *Oga*. These findings establish *Oga* as a critical regulator of Gal-3 expression and systemic abundance. The data reveal a mechanistic link between *O*-GlcNAc signaling enzyme *Oga*, and lectin-mediated metabolic inflammation, suggesting that *Oga* activity influences Gal-3 homeostasis and may affect its interpretation as a biomarker in metabolic disease.

## 1. Introduction

*O*-GlcNAcylation, the noncanonical post-translational modification (PTM) form of protein glycosylation, involving the attachment of *O*-GlcNAc to serine and threonine residues, has emerged as a major cellular signaling mechanism, rivaling protein phosphorylation in the breadth of its target proteins [1,2,3]. *O*-GlcNAc modifications have been identified on over 15,000 proteins across 43 distinct species [4,5]. The addition and removal of *O*-GlcNAc on nucleocytoplasmic and mitochondrial proteins is catalyzed by the intracellular enzymes *O*-GlcNAc transferase (*Ogt*) and *Oga* respectively [6,7]. As a dynamic PTM, *O*-GlcNAcylation regulates essential cellular processes such as gene expression, signal transduction, the cell cycle, nutrient sensing, protein homeostasis, cellular responses to diverse stress conditions, and epigenetic regulation [8,9,10,11,12,13,14,15,16,17]. Additionally, *O*-GlcNAcylation contributes to diverse biological processes by modulating protein dynamics including synthesis, localization, degradation, and interaction with other macromolecules [3,6,14,15,17,18,19,20,21]. Altered and abnormal *O*-GlcNAcylation is frequently observed in multiple disease conditions including cancer, cardiac hypertrophy, Alzheimer’s disease, renal disease, and type 2 diabetes [22,23,24,25,26,27,28]. This supports the notion that *O*-GlcNAcylation is implicated not only in maintaining normal cellular functions but also in the pathological processes of human diseases [3,29].

Gal-3, encoded by the *Lgals3* gene, has a high affinity for β-galactoside-containing glycans, particularly galactose β-(1,4)-*N*-acetylglucosamine (*N*-acetyllactosamine) linkages found in both *N*-linked and *O*-linked glycans, glycolipids, and blood group antigens. In mammals, Gal-3 is ubiquitously distributed throughout the body and is expressed in various tissues and cell types, except for the spleen and lymph nodes. Gal-3 is expressed both intracellularly (nucleus, cytoplasm) and extracellularly (cell surface, extracellular space) and is highly expressed in inflammatory and epithelial cells as well as in immune cells like macrophages, dendritic cells, and neutrophils. Gal-3 is also expressed in various tissues and organs, including the kidney, heart, blood vessels, skin, bones, and nervous system [30]. This dynamic distribution is thought to enable Gal-3 to participate in diverse cellular functions such as gene regulation, mRNA splicing, glucose homeostasis, and signaling pathways linked to cell survival and apoptosis [31]. Since Gal-3 is readily secreted to the cell surface as well as in biological fluids such as blood and urine, particularly from injured or inflammatory cells, it holds potential as a sensitive diagnostic and prognostic indicator for a range of pathological conditions, including metabolic syndromes like obesity, cancer, heart failure, fibrosis, and inflammatory diseases such as rheumatoid arthritis and chronic kidney disease [32,33]. Gal-3 has also garnered considerable attention for its role in the onset and progression of long-term diabetes complications because of its ability to bind advanced glycation end products (AGEs) and advanced lipoxidation end products (ALEs) that accumulate in target organs, triggering proinflammatory and prooxidant pathways that contribute to tissue damage [34,35].

Surprisingly, *O*-GlcNAcylation and Gal-3 exhibit a unique interplay, leading to distinct interactions and functions that make them a compelling subject for further study [30]. *O*-GlcNAcylation and Gal-3 share a similar subcellular localization [30]. Functionally, they are at the crossroads of nutrient sensing through the hexosamine biosynthetic pathway (HBP) and UDP-GlcNAc availability, as well as associated with an overlapping spectrum of pathological conditions [30,36]. Previous reports have indicated that the functional relationship between O-GlcNAcylation and Gal-3 expression appears to be highly context-dependent, varying with cell type, differentiation state, and microenvironmental conditions. Pharmacological manipulation of *O*-GlcNAc cycling enzymes differentially affects Gal-3 expression depending on cell type and context. In MCF7 breast cancer cells, both *Oga* inhibition (TMG) and *Ogt* inhibition (Ac-5SGlcNAc) altered *Lgals3* transcript levels, whereas HL-60 leukemia cells showed no such response under basal conditions [37]. However, during ATRA-induced neutrophilic differentiation of HL-60 cells, global *O*-GlcNAc levels dropped significantly and Gal-3 was markedly upregulated, with the strongest effect observed under serum-free conditions [38]. This pattern suggests that differentiation-associated remodeling of *O*-GlcNAcylation can override the transcriptional unresponsiveness seen in undifferentiated HL-60 cells.

A broader theme emerging across multiple model systems is that *O*-GlcNAc levels act as a rheostat governing galectin-3 trafficking between intracellular and secretory compartments. In stem cells, high *O*-GlcNAc promotes intracellular galectin retention, while low *O*-GlcNAc in extraembryonic endoderm cells correlates with enhanced secretion and differentiation [39]. Proteomic analysis of the glioblastoma secretome further identified galectin-3 as a likely substrate for *O*-GlcNAc modification [40]. Together, these findings across cancer, leukemia, and stem cell models indicate a conserved yet context-sensitive mechanism by which *O*-GlcNAcylation regulates Gal-3 expression, localization, and secretion. These findings have broad implications for tumor biology and cellular differentiation. We have previously reported that Gal-3 is a substrate for *Ogt* and that secreted Gal-3 levels are tightly regulated by *O*-GlcNAc cycling [36]. To further investigate the role of *O*-GlcNAc dynamics on Gal-3 secretion in vivo, we examined circulating Gal-3 levels in plasma and serum as well as *Lgals3* gene expression in *Oga* WT, HET, and KO mice.

## 2. Materials and Methods

### 2.1. Mouse Maintenance and Diet

The male and female mice for *Oga* WT, KO and HET experiments (C57BL/6J) were maintained according to the animal protocol #K023-LCBB-23 dated 23 September 2023 and maintained on a standard mouse chow diet (fat: 15% kcal; energy density: 3.1 kcal/g; NIH-07 diet #7022, Envigo, Indianapolis, IN, USA). Male C57BL/6NTAC mice utilized for obesity model experiments were maintained according to the animal protocol #K083-LBC-23 dated 1 September 2023. One cohort was maintained on a standard mouse chow diet. A separate cohort of 8-week-old male littermates were switched to a high-fat diet (HFD, fat: 60% kcal; energy density: 5.49 kcal/g; #F3282, BioServ, Flemington, NJ, USA) for 8 weeks and body weight was recorded weekly. All animal experiments were performed between 16 and 20 weeks of age, and they were housed under a 12 h light cycle. All animal procedures were approved by the Institutional Animal Care and Use Committee of the National Institute of Diabetes and Digestive and Kidney Diseases (NIDDK) and conducted in accordance with the U.S. National Institutes of Health (NIH) Guidelines for Animal Research. The animals were randomized for the study, and the investigators were blinded on the genotypes but not the sex differences.

### 2.2. Blood Profiling

Lean and obese mice were assigned to either ad libitum feeding or a 12–14 h fasting, after which blood glucose levels were measured under both fed and fasted conditions using a portable glucometer (Contour, Ascensia Diabetes Care US, Inc., Parsippany-Troy Heals, NJ, USA). Blood was collected between 8 am and 10 am from the tail vein of lean and obese mice, and facial blood was collected from *Oga* WT, KO and HET mice in serum collection tubes (KMIC-SER, Kent Scientific Corporation, Torrington, CT, USA) or chilled EDTA K containing plasma collection tubes (KMIC-EDTA Kent Scientific Corporation, Torrington, CT, USA). Blood was centrifuged at 4 °C for 10 min at 2000× *g* to obtain serum or plasma and stored at −80 °C until use. Mouse Gal-3 ELISA kit (ab 203369, Abcam, Waltham, MA, USA) was used to measure plasma and serum Gal-3 levels following the manufacturer’s instructions. Serum and plasma samples were diluted in 1:1000 ratio into sample dilution buffer for ELISA assay.

### 2.3. RT-qPCR

At least 10 mg of tissue was collected for each sample. RNA was extracted using RNeasy plus micro kit (74104, Qiagen, Germantown, MD, USA) following manufacturer’s protocol. cDNA was prepared from 1 μg of RNA using cDNA synthesis kit (95047-100, Quantabio, Beverly, MA, USA) following manufacturer’s protocol. Fast SYBR Green Master Mix (4385612, Applied Biosystems, CA, USA) was used for amplification following manufacturer’s protocol using 5 ng of cDNA and the appropriate primer (Supplemental Table S1) on a 7900HT fast real-time PCR system (4351405, Applied Biosystems, Foster City, CA, USA). Each reaction was performed in technical triplicate. Relative gene expression was normalized to the geometric mean of *Rplp0*.

### 2.4. Statistical Analysis

Prism (version 11.0.2 for MacOS, GraphPad, San Diego, CA, USA) was used for all statistical analysis. A two-way ANOVA test was used to determine significance as indicated in figure legends. *p*-values are shown in graph and error bars represent the standard deviation centered on the mean.

## 3. Results

### 3.1. Confirmation That Elevated Circulating Gal-3 Is Linked to Obesity and Type 2 Diabetes

**Figure 1:**
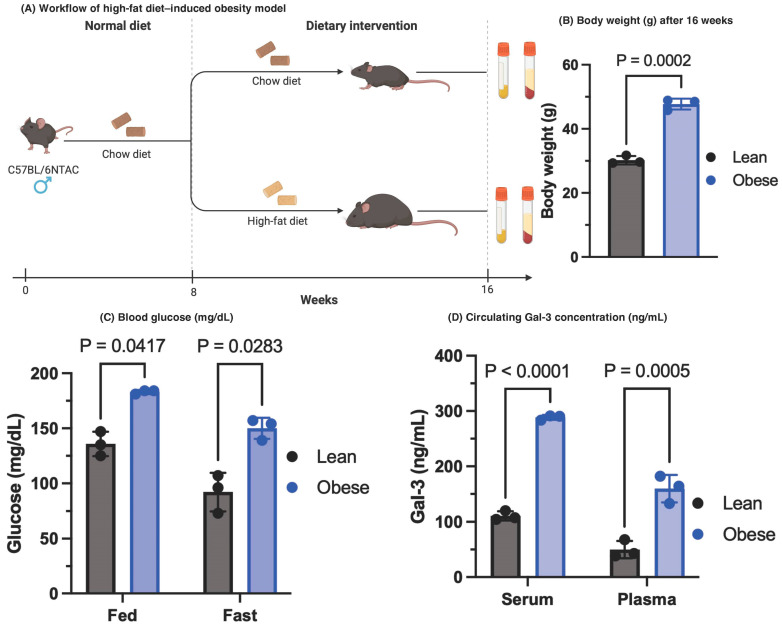
Serum and plasma Gal-3 levels are elevated in obese mice. (**A**) Workflow representing the regular and HF dietary intervention of the C57BL/6NTAC mice. (**B**) Body weight of regular and HFD-induced mice. Welch’s *t*-test was performed. (**C**) Fed and fasting blood glucose of regular and HFD-induced mice shows higher blood glucose for obese mice. Blood glucose levels were measured using commercially available glucose strips and portable glucometer. (**D**) Serum and plasma Gal-3 levels of obese mice are higher than that of the lean mice. N = 3 biological replicates. Circulating Gal-3 levels were measured using ELISA. An ordinary 2-way ANOVA test was performed. *p*-values are shown in the graph and error bars represent the standard deviation centered on the mean.

In human studies, circulating Gal-3 levels have been reported to correlate positively with obesity and type 2 diabetes [41,42], although its precise role remains unclear, with some evidence suggesting that Gal-3 may act as a marker of glucose disposal [41,43,44,45,46], or insulin sensitivity [47]. In animal studies, macrophage Gal-3 expression is elevated in mice fed high-fat diet, falling when mice are returned to a normal diet [48], and plasma Gal-3 levels are elevated in HFD-induced obese mice [49]. To further investigate this in our in-house mouse colonies, male C57BL/6NTAC mice were fed a HFD for 8 weeks (Figure 1A). Body weight (Figure 1B) as well as both fed and fasting blood glucose levels (Figure 1C) were measured to confirm the establishment of diet-induced obesity. Circulating Gal-3 levels were then quantified using ELISA in serum and plasma from lean and obese mice (Figure 1D). Both serum and plasma Gal-3 concentrations were nearly threefold higher in obese mice compared with lean controls (Figure 1D), indicating that diet-induced obesity markedly enhances Gal-3 production and/or release into circulation. These findings parallel human studies [41,46,50] where elevated Gal-3 levels are associated with obesity and type 2 diabetes, thereby underscoring the potential of Gal-3 as a prognostic indicator of metabolic disease and possible contributor to insulin resistance through metabolic and inflammatory stress-responsive pathways [41,42,48].

### 3.2. The Distribution and Levels of Gal-3 Are Influenced by Oga Expression

**Figure 2:**
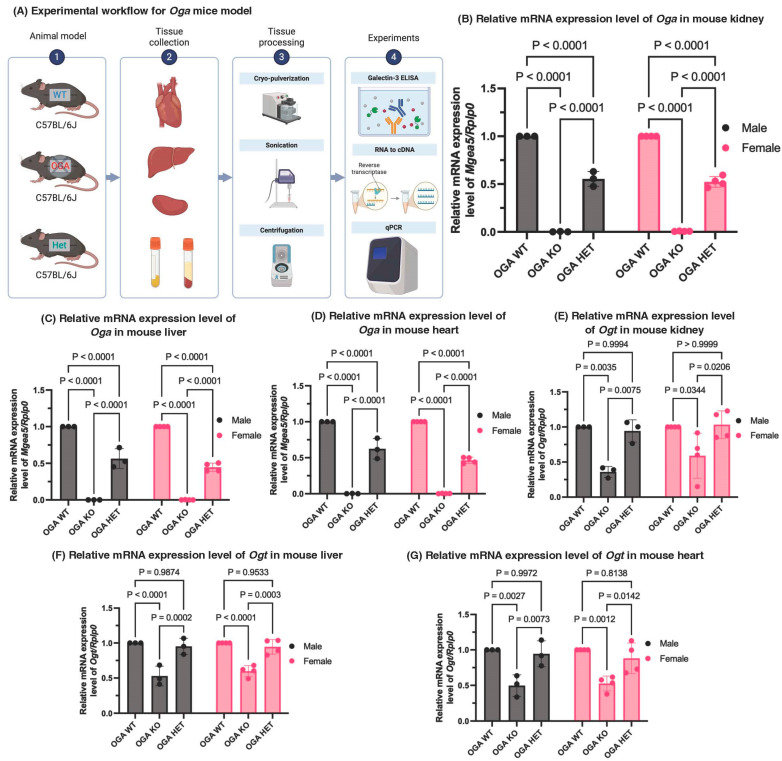
Genotyping of *Oga* WT, KO and HET mice organs and *Ogt* mRNA expression levels in *Oga* WT, KO and HET mice organs. (**A**) Schematic presentation of the experimental workflow. (**B**–**D**) *Oga* mRNA expression levels in the kidney (**B**), liver (**C**), and heart (**D**) tissues of the *Oga* WT, KO, and HET mice. *Ogt* mRNA expression levels in the kidney (**E**), liver (**F**), and heart (**G**) tissues of the *Oga* WT, KO, and HET mice. N = 3 and 4 biological replicates for male and female mice respectively. Relative mRNA expressions were measured using qPCR. An ordinary 2-way ANOVA test was performed. *p*-values are shown in the graph and error bars represent the standard deviation centered on the mean.

Earlier studies from our laboratory as well as others have linked *O*-GlcNAc cycling to stress response [16,51], nutrient sensitivity [17,27,52], and insulin resistance [27,53,54,55,56]. Systematic studies utilizing mouse embryonic fibroblasts (MEFs) derived from *Oga* WT, HET and KO mice showed insulin response signaling through GSK3β is dramatically altered in *Oga* KO animals and *Oga* KO neonatal mice exhibit morbid hypoglycemia [56]. Our previous exploration of the role of *O*-GlcNAc cycling in Gal-3 secretion has been focused on MEFs derived from *Oga* WT and KO mice [36]. However, the influence of *O*-GlcNAc cycling enzyme *Oga* in modulating Gal-3 distribution in vivo, especially in mouse blood and tissues, remains uncharacterized. In order to study the impact of *O*-GlcNAc cycling enzyme *Oga* on tissue-specific and circulating Gal-3 levels in the blood in vivo, we utilized male and female mice in which the *Oga* gene had been deleted globally [56]. For this study, facial blood was collected from 16- to 20-weeks-old *Oga* WT, HET, and KO mice to obtain serum and plasma. The mice were then euthanized to separately harvest the heart, liver, and kidney (Figure 2A). Tissue-specific *Oga* mRNA levels of those whole body *Oga* WT, HET, and KO mice were measured to validate the attenuated *Oga* expression levels in the kidney (Figure 2B), liver (Figure 2C), and heart (Figure 2D). Further measurement of *Ogt* mRNA expression, the enzyme responsible for adding *O*-GlcNAc, in the kidney (Figure 2E), liver (Figure 2F), and heart (Figure 2G) suggested altered *O*-GlcNAcylation levels in these mice.

**Figure 3:**
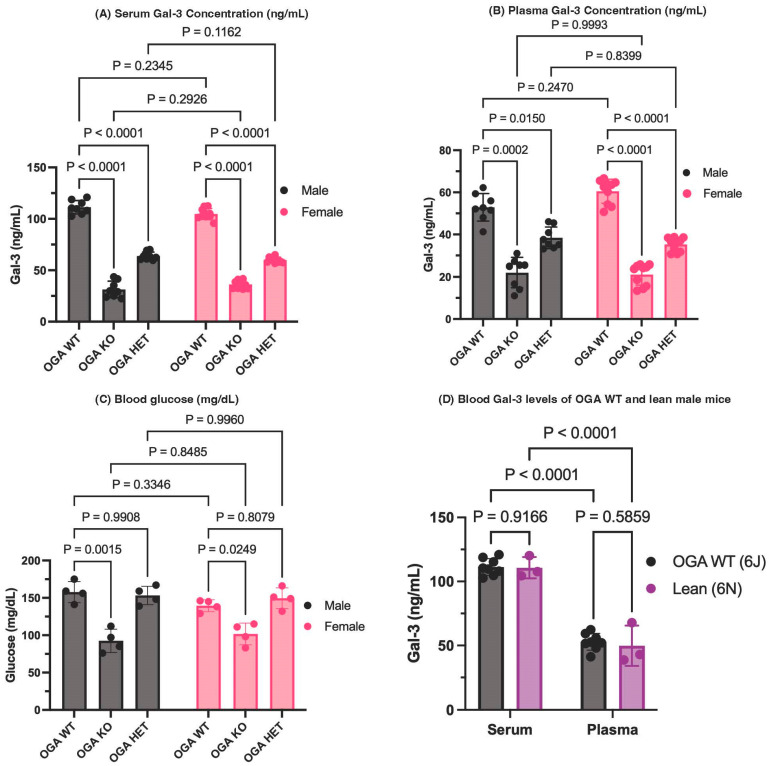
Serum and plasma Gal-3 levels are significantly lower in *Oga*-depleted mice. (**A**) Serum Gal-3 levels of *Oga* KO male and female mice are lower than *Oga* WT, and HET mice exhibit an intermediate level. (**B**) Plasma Gal-3 levels of *Oga* KO male and female mice are lower than *Oga* WT, and HET mice exhibit an intermediate level. N = 8 and 10 biological replicates for male and female mice respectively. (**C**) Blood glucose levels in *Oga* KO male and female mice are significantly lower than *Oga* WT, and HET mice exhibit similar blood glucose levels to WT mice. N = 4 biological replicates. (**D**) Both *Oga* WT and lean male mice exhibit similar serum and plasma Gal-3 concentrations. N = 8 and 4 biological replicates for *Oga* WT and lean mice respectively. Blood glucose levels were measured using commercially available glucose strips and portable glucometer. Circulating Gal-3 levels were measured using ELISA. An ordinary 2-way ANOVA test was performed. *p*-values are shown in the graph and error bars represent the standard deviation centered on the mean.

ELISA was used to measure the amount of circulating Gal-3 in both male and female mouse blood. There is a significant decrease in the Gal-3 concentration in both serum (Figure 3A) and plasma (Figure 3B) in male and female *Oga* KO mice relative to the WT controls. *Oga* HET mice exhibit intermediate Gal-3 levels. Notably, Gal-3 concentrations (ng/mL) are elevated in the serum relative to the plasma in both male and female mice, further indicating that Gal-3 levels are influenced by blood coagulation processes. Blood glucose levels measured under fed conditions revealed that *Oga* KO mice were significantly hypoglycemic (Figure 3C), consistent with our earlier observations in neonatal mice [56]. *Oga* HET mice did not show this effect. Together, this observation, along with the positive correlation between blood Gal-3 concentrations and *Oga* expression, suggests that circulating Gal-3 levels are not solely determined by glucose availability, but are critically influenced by *Oga* and perhaps the extent of *O*-GlcNAcylation.

Additionally, comparison of basal Gal-3 concentrations in serum and plasma (ng/mL) revealed no significant differences between *Oga* WT males (C57Bl/6JMgea5^tm2Jah^/cre) and lean control males (C57BL/6NTAC), indicating that genotype alone does not alter circulating Gal-3 levels under baseline conditions (Figure 3D). This underscores the idea that the elevation of Gal-3 observed in obese mice arises primarily from diet-induced metabolic stress rather than from inherent genetic background and suggests that *Oga* expression level may contribute to the regulation of circulating Gal-3 through mechanisms that remain to be clarified and warrant further investigation.

**Figure 4:**
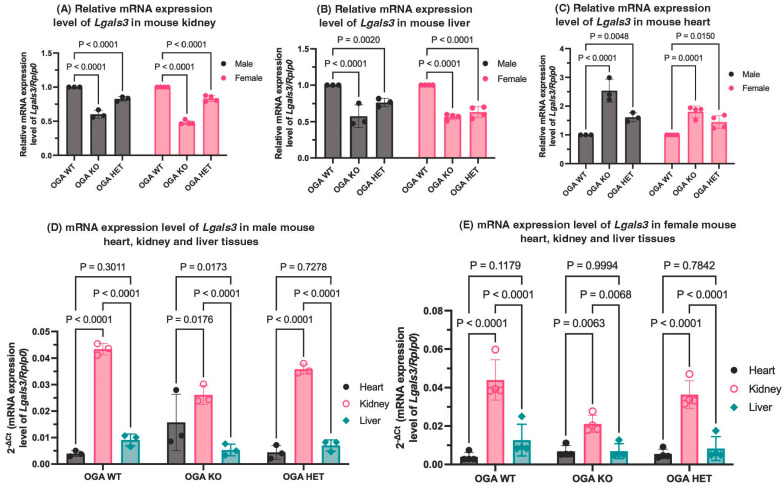
*Lgals3* mRNA is differentially expressed in different tissues. *Lgals3* mRNA expression is higher in *Oga* WT mouse liver (**A**) and kidney (**B**) compared to Oga KO mouse liver and kidney tissues. (**C**) *Lgals3* mRNA expression is lower in *Oga* WT mouse heart compared to *Oga* KO mouse heart tissue. Comparison of *Lgals3* mRNA expression in different organs of (**D**) male and (**E**) female *Oga* WT, KO, and HET mice. N = 3 and 4 biological replicates for male and female mice respectively. Relative mRNA expression was measured using qPCR. An ordinary 2-way ANOVA test was performed. The *p*-values are shown in the graph and error bars represent the standard deviation centered on the mean.

To further assess the relationship between *Oga* expression and Gal-3 regulation, we quantified tissue-specific *Lgals3* transcript levels using RT-qPCR in kidney, liver, and heart tissues obtained from *Oga* WT, HET, and KO mice. In contrast to circulating Gal-3 concentrations in mouse plasma or serum, tissue-specific *Lgals3* expression exhibited organ-dependent variation in response to altered *Oga* expression. In the kidney (Figure 4A) and liver (Figure 4B), *Lgals3* transcript levels were significantly downregulated in both male and female *Oga* KO mice compared with WT controls, whereas HET mice exhibited intermediate expression. Contrastingly, in heart tissue (Figure 4C) from both sexes, *Lgals3* expression was significantly elevated in *Oga* KO mice relative to the WT controls, with HET mice again displaying intermediate levels. Together with circulating Gal-3 measurements, these findings indicate that the loss of *Oga* modulates *Lgals3* expression in an organ-dependent manner, consistent with tissue-specific roles of *O*-GlcNAc cycling in the regulation of Gal-3 expression. Additionally, these findings suggest that altered *O*-GlcNAc homeostasis maintained through *Ogt*-*Oga* axis, may differentially influence Gal-3-mediated processes such as inflammation, fibrosis, and cardiac remodeling across tissues. The analysis of relative *Lgals3* transcript levels across kidney, liver, and heart tissues from *Oga* WT, HET, and KO mice revealed their differential contribution to the overall Gal-3 pool. Among the tissues examined, the kidney exhibited the highest *Lgals3* expression, the heart displayed the lowest, and the liver showed an intermediate level corresponding to their relative contributions to the overall Gal-3 pool (Figure 4D,E), and corroborating the established tissue distribution of *Lgals3* RNA expression [30].

## 4. Discussion

In this study, we report distinct alterations in circulating Gal-3, a recognized prognostic indicator, in two complementary mouse models: diet-induced type 2 diabetic obesity and *Oga*-deficient mice. In the dietary model of obesity, both serum and plasma Gal-3 concentrations (ng/mL) were significantly elevated relative to lean controls, consistent with clinical observations linking increased Gal-3 to obesity and diabetes [41,42,46,50]. This increase likely reflects enhanced Gal-3 production and/or release in response to metabolic and inflammatory stress associated with obesity. In contrast, *Oga* KO mice exhibited significantly reduced circulating Gal-3 levels compared with *Oga* WT littermates, whereas *Oga* HET mice displayed intermediate levels, suggesting a gene dosage effect. These findings point toward a role for the *O*-GlcNAc cycling enzyme *Oga* in regulating Gal-3 homeostasis, where loss of *Oga* may impair pathways that normally sustain basal Gal-3 expression and secretion. Tissue-specific Gal-3 mRNA (*Lgals3*) expression is also significantly altered in *Oga*-altered mice. Together, these contrasting models highlight the dual nature of Gal-3 as both a prognostic indicator and potential effector of metabolic dysfunction, reinforcing its relevance to the pathophysiology of obesity and type 2 diabetes.

*O*-GlcNAcylation has been implicated in altering the functional activity of proteins and regulating their intracellular trafficking and localization [18,57]. *O*-GlcNAcylation directly modifies key subunits of the protein complexes responsible for the formation of COPII, COPI, and clathrin-coated vesicles (CCVs), as well as other proteins involved in vesicle budding and fusion [18,57]. Additionally, *O*-GlcNAcylation has been reported to regulate proteins involved in autophagy and noncanonical secretory pathways [58,59,60]. Gal-3 and *O*-GlcNAcylation are also closely interconnected at both structural and functional levels, particularly in their roles within intracellular trafficking and secretion. The translocation of Gal-3 to the nucleolus is dependent on the importin transport complex [61], structurally resembling the enzyme *Oga* involved in the *O*-GlcNAcylation cycle [62]. Notably, Gal-3 is secreted via a “noncanonical” secretory pathway [30,63,64], a process that is significantly influenced by the dynamics of *O*-GlcNAcylation [18,57,60]. This interplay suggests that fluctuations in the *O*-GlcNAc cycling enzyme *Oga* not only affect intracellular trafficking of Gal-3 but may also influence its extracellular functions in cell signaling, immunity, and disease pathology. Previous studies have shown that the expression and secretion of Gal-3 in HL-60 cells is influenced by the homeostatic regulation of intracellular protein *O*-GlcNAcylation levels. Furthermore, our laboratory has previously demonstrated that Gal-3 undergoes *O*-GlcNAcylation and that cellular *O*-GlcNAcylation levels modulate Gal-3 secretion in cultured cells [36].

A few studies have investigated the relationship between circulating levels of Gal-3, obesity and parameters of glucose metabolism, as well as insulin sensitivity in patients with diabetes. Weigert et al. reported that circulating Gal-3 levels were elevated in 30 overweight nondiabetic individuals and in 30 patients with type 2 diabetes compared with 23 normal-weight controls [45]. Moreover, Gal-3 concentrations correlated positively with body mass index (BMI), and in a BMI-dependent manner with leptin, resistin, IL-6, and age [45]. In a clinical cohort of 174 individuals stratified into diabetic, prediabetic, and nondiabetic groups, and blood Gal-3 concentrations were significantly elevated in diabetic patients compared with both prediabetic and nondiabetic subjects and were also higher in prediabetic individuals than in the nondiabetic group [46,50]. In contrast, a smaller study involving 20 individuals suggested that Gal-3 may influence insulin concentrations more strongly than glucose levels, raising the possibility that increased Gal-3 activity in diabetic subjects could improve insulin sensitivity [47]. However, given the limited cohort size, these observations should be interpreted with caution and require validation in larger populations. While these two studies appear to be contrasting, their differences can be explained if Gal-3 upregulation acts as an adaptive mechanism that counteracts the progression of metabolic derangement by promoting glucose disposal. From this perspective, Gal-3 levels may rise with the development of obesity and diabetes, thereby serving as a marker of these disorders, while potentially exerting a protective influence against insulin resistance. This interpretation is consistent with the proposed role of Gal-3 in facilitating the clearance of AGEs and ALEs [43,44]. Supporting this notion, several studies in nondiabetic individuals have demonstrated that circulating levels of AGEs or their reactive carbonyl precursors are independently correlated with insulin resistance, as assessed using the HOMA-IR index [65].

*Oga* activity serves as a critical node linking nutrient sensing, autophagy regulation, and glucose metabolism through its role in dynamically removing *O*-GlcNAc modifications from target proteins. The hexosamine biosynthetic pathway (HBP), which produces the *O*-GlcNAc donor substrate UDP-GlcNAc, drives increases in *O*-GlcNAc levels that may function as a real-time readout of cellular nutrient status [66]. Under conditions of glucose abundance, elevated HBP flux sustains high *O*-GlcNAcylation, which suppresses autophagy in part by modulating mTORC1 and AMPK activity, both of which are subject to *O*-GlcNAc modification. *O*-GlcNAcylation of AMPK impairs its ability to phosphorylate TSC1/2, thereby sustaining mTORC1 activation and restraining autophagic initiation [67,68]. Conversely, increased *Oga* activity or nutrient deprivation that reduces HBP flux lowers global *O*-GlcNAc levels and permits autophagy induction, with calcium-dependent *O*-GlcNAc signaling shown to drive hepatic autophagy during starvation [69]. Reduced mTORC1 activity downstream of these events promotes dephosphorylation and nuclear translocation of TFEB, the master transcriptional regulator of lysosomal biogenesis and autophagic gene programs [70]. Furthermore, disruption of *O*-GlcNAc cycling through *Oga* inhibition impairs mitophagy by interfering with *O*-GlcNAcylation of PINK1, a modification that, upon mitochondrial damage, stabilizes and activates PINK1 to initiate selective mitochondrial degradation [71]. This PINK1-dependent mechanism directly couples glucose uptake and HBP flux to mitochondrial quality control, establishing a bidirectional relationship by which glucose metabolism regulates mitophagy, and mitophagy in turn influences oxidative metabolism [71].

Within this framework, the reduction in circulating Gal-3 observed in *Oga* KO mice raises the possibility that disruption of *O*-GlcNAc cycling may modify or limit this potential protective function, thereby influencing the relationship between metabolic stress and insulin sensitivity. *Oga* KO mice display a striking constellation of metabolic and inflammatory abnormalities reflecting systemic dysregulation of nutrient sensing and stress signaling. Complete loss of *Oga* activity leads to chronically elevated *O*-GlcNAc levels and broad physiological disruption, including impaired growth, altered energy homeostasis, and signs of organismal stress [72,73]. Metabolically, these mice exhibit pronounced hypoglycemia even under fed conditions, indicating defective glucose regulation and inappropriate insulin or hepatic metabolic responses [56]. These *Oga* KO mice showed semi-penetrant perinatal lethality accompanied by low circulating glucose and depleted liver glycogen, indicating severe failure of early metabolic adaptation [56]. Adult *Oga* HET and KO mice accumulated more fat mass than wild-type, which was further exacerbated by high-fat feeding. Females showed reduced insulin sensitivity, impaired glucose tolerance, hyperleptinemia, and a higher respiratory exchange ratio, indicating preferential use of carbohydrate over lipid as an energy source [56]. *Oga* activity and proper *O*-GlcNAc cycling are essential for maintaining whole body metabolic homeostasis and preventing obesity and insulin resistance. Disruption of *O*-GlcNAc cycling is closely tied to aberrant inflammatory phenotypes, as *O*-GlcNAc dysregulation broadly modulates immune cell activation and cytokine responses, linking metabolic imbalance to altered immune states [56]. *O*-GlcNAcylation tunes innate and adaptive immune signaling: NF-κB activation, cytokine production, T-cell receptor signaling, and macrophage polarization. Altered *O*-GlcNAc cycling can skew immune responses, promoting chronic low-grade inflammation or impaired defense [56].

Future studies are needed to determine the precise mechanisms by which *O*-GlcNAc cycling regulates Gal-3 and to clarify whether this pathway contributes directly to the development of insulin resistance.

## 5. Conclusions

This study demonstrates that *Oga* is a key regulator of Gal-3 expression and systemic abundance in vivo. HFD-induced obesity markedly elevates circulating Gal-3, whereas the genetic loss of *Oga* produces a gene-dosage-dependent reduction in Gal-3 in both serum and plasma, accompanied by tissue-specific changes in *Lgals3* transcription. These findings establish *O*-GlcNAc cycling as a previously unrecognized control point for Gal-3 homeostasis in vivo. By linking nutrient-sensitive *O*-GlcNAc signaling to Gal-3-mediated inflammatory and metabolic pathways, this work provides a mechanistic framework explaining how metabolic stress and post-translational modification intersect to regulate a clinically important biomarker, with implications for the interpretation of Gal-3 in obesity, diabetes, and related disorders.

## Data Availability

The original contributions presented in this study are included in the article/Supplementary Materials. Further inquiries can be directed to the corresponding authors.

## Supplementary Materials

The following supporting information can be downloaded at: https://www.mdpi.com/article/10.3390/cells15131181/s1, Table S1: Primer sequence of Lgals3, Mgea5, and RPLP0 used for RT-qPCR experiment.

## Abbreviations

The following abbreviations are used in this manuscript:

Gal-3	Galectin-3
O-GlcNAc	*O*-linked β-*N*-acetylglucosamine
OGA	*O*-GlcNAse
WT	Wild-type
HET	Heterozygous
KO	Knockout
PTM	Post-translational modification
HFD	High-fat diet
OGT	*O*-GlcNAc transferase
mRNA	Messenger RNA
ELISA	Enzyme-linked immunosorbent assay
RT-qPCR	Reverse transcription quantitative polymerase chain reaction
AGEs	Advanced glycation end products
ALEs	Advanced lipoxidation end products
COP	Coat protein complex
CCV	Clathrin-coated vesicles
TMG	Thiamet G
Ac-5SGlcNAc	Per-*O*-acetylated 5-thio-N-acetylglucosamine
mTROC1	Mammalian or mechanistic target of Rapamycin complex 1
TFEB	Transcription factor EB
PINK1	PTEN induced putative kinase 1
AMPK	AMP-activated protein kinase
TSC1/2	Tuberous sclerosis complex 1 and 2
