# Supplementary material for: Perturbing O-GlcNAcase Modulates the Expression and Distribution of Galectin-3
Source: Cells. 2026 Jun 29;15(13):1181. doi: 10.3390/cells15131181 (PMC13360182; doi:10.3390/cells15131181)
Supplement: Supplementary file 1 [file cells-15-01181-s001.zip › cells-4123480-supplementary.pdf]

Supporting Table S1

Primer Name (Mouse)	Sequence (3’-5’)
Lgals3 Forward	AACACGAAGCAGGACAATAACTGG
Lgals3 Reverse	GCAGTAGGTGAGCATCGTTGAC
Mgea5 Forward	GTG CAG TGG TTA GGG TGT CG
Mgea5 Reverse	AGC AAA CGC TGG AAC TCT CC
Rplp0 Forward	GCTTCGTGTTCACCAAGGAGGA
Rplp0 Reverse	GTCCTAGACCAGTGTTCTGAGC

Supporting Table S1: Primer sequence of *Lgals3, Mgea5, and RPLP0 used* for RT-qPCR experiment
